# A Pilot 24-Week ‘Bulk and Cut’ Dietary Protocol Combined with Resistance Training Is Feasible and Improves Body Composition and TNF-α Concentrations in Untrained Adult Males

**DOI:** 10.3390/nu17071265

**Published:** 2025-04-04

**Authors:** Anthony J. Giannopoulos, Steve Kottaras, Bryan Allanigue, Jeremia M. Coish, David S. Ditor, Val A. Fajardo, Panagiota Klentrou

**Affiliations:** 1 Department of Kinesiology, Brock University, St. Catharines, ON L2S 3A1, Canadastevenkottaras@gmail.com (S.K.); dditor@brocku.ca (D.S.D.); vfajardo@brocku.ca (V.A.F.); 2 Core Health Collective, St. Catharines, ON L2R 5J3, Canada; 3 Department of Health Sciences, Brock University, St. Catharines, ON L2S 3A1, Canada

**Keywords:** exercise and diet intervention, obesity, blood lipids, inflammation

## Abstract

**Background/Objectives**: This study piloted a 24-week bodybuilding program combining resistance training (RT) with a dietary bulk-and-cut protocol in middle-aged adult males. **Methods**: Seven untrained males (33 ± 3.0 years; BMI = 35.0 ± 4.6 kg/m^2^; body fat = 36 ± 5%) completed a 24-week intervention combining RT with a dietary protocol consisting of 12-week cycles of caloric bulking (0–12 weeks) and cutting (12–24 weeks). The participant retention rate was 64%, while compliance with training was 96.7%, and adherence to dietary cycles was over 93%. To assess the preliminary efficacy of the intervention, venous blood samples and measurements of body composition (BodPod), muscle strength, and VO_2_max (cycle ergometer) were collected at baseline (week 0) and following the bulking (week 12) and cutting (week 24) cycles. Circulating lipids (triglycerides, total, low-density, and high-density cholesterol), C-reactive protein (CRP), tumor necrosis factor-alpha (TNF-α), interleukin-6 (IL-6), and interleukin-10 (IL-10) were measured in serum. **Results**: The training led to significant increases in muscle strength, especially in the deadlift (+46%, *p* < 0.001) and squat (+65%, *p* < 0.001). Improvements in body composition were characterized by an increase in fat-free mass and a decrease in body fat percentage over the 24-week intervention (+3% and −6%, respectively, *p* < 0.05). Lipids, CRP, IL-6, and IL-10 did not change significantly, but there was a notable reduction in TNF-α (time effect *p* = 0.05, _p_η^2^ = 0.39), with 15% lower concentrations at week 24 compared to baseline, indicating reduced inflammation. **Conclusions**: Overall, the pilot intervention achieved high compliance and adherence rates, leading to improvements in body composition and lower resting TNF-α concentrations in a group of middle-aged males with obesity.

## 1. Introduction

The obesity epidemic has partly stemmed from increased calorie consumption and reduced physical activity levels [1]. Resistance training, which includes strength and power exercises, has garnered interest for its role in maintaining a healthy body weight and overall health. Evidence has shown that this type of training has been shown to enhance muscle strength and mass, reduce body fat and blood pressure, lower low-density lipoprotein (LDL) levels, increase high-density lipoprotein (HDL) levels, help prevent osteoporosis, and reduce the risk of type II diabetes and all-cause mortality [2,3,4,5]. Despite these documented benefits of resistance training, many people tend to favor aerobic exercises or high-intensity interval training when aiming to lose weight and reduce fat mass.

In contrast, competitive bodybuilders focus on resistance training and building lean mass to reduce fat mass. They often follow a dietary protocol that includes a bulking cycle (i.e., high caloric and protein intake) followed by a cutting cycle (i.e., reduced caloric intake with maintained protein intake). This approach seems to help them gain lean mass while lowering fat mass [6,7]. Indeed, competitive bodybuilders would typically cut their caloric intake for two to four months while increasing energy expenditure to become as lean as possible before competition [6,7,8]. Although there is no consensus on the optimal carbohydrate and fat intake for competitive bodybuilding, a protein intake of 1.2–1.7 g/kg/day is recommended [8,9]. However, while this level of protein is required in competitive, resistance-trained individuals, recent studies have shown protein intakes exceeding ~1.6 g/kg/day do not lead to additional increases in muscle mass for untrained and overweight individuals [10,11].

Despite the potential benefits of this bodybuilding approach combining resistance training with diet to improve body composition, there is no evidence regarding its effect on inflammation. Diet plays a significant role in regulating the immune response. Malnutrition can cause immunosuppression, increasing susceptibility to infections, while overnutrition can lead to immunoactivation, making individuals more prone to inflammatory conditions [12,13]. In obesity, chronic low-grade inflammation is recognized as a major risk factor for the progression of insulin resistance, type II diabetes, atherosclerosis, and certain types of cancer [14,15,16]. Furthermore, select cytokines are commonly observed to increase immediately following both resistive, aerobic, or combined exercise, with IL-6 exhibiting the largest increase [17,18,19]. However, the acute rise in inflammatory cytokines from exercise is not thought to promote chronic inflammation. Instead, the repeated increase in inflammatory markers following repeated resistive, aerobic, or combined exercise sessions is believed to contribute to a decrease in chronic low-grade inflammation over the training period [18,19].

While it is uncertain whether this combined resistance training and dietary bulk-and-cut protocol is feasible and beneficial for untrained individuals, exploring this method could provide a viable alternative for weight loss and overall health improvements in the general population. Therefore, we designed a pilot study to examine the feasibility and effects of a novel 24-week resistance training intervention combined with a dietary protocol, consisting of bulk and cut cycles, on body composition, blood lipids (triglycerides, total cholesterol, HDL, and LDL) and inflammatory markers, including C-reactive protein (CRP), tumor necrosis factor-alpha (TNF-α), interleukin 6 (IL-6), and the anti-inflammatory interleukin (IL-10) in untrained, middle-aged adult males. To confirm the preliminary efficacy of this newly designed intervention, secondary outcomes also included muscle strength and VO_2_max (cycle ergometer).

## 2. Materials and Methods

### 2.1. Participants

A total of 12 adult males, aged 25–45 years, free from any chronic conditions, and nonsmokers were recruited to participate in this pilot study. One of those dropped out before starting the intervention, thus attaining a final sample of 11 participants (mean age: 33.0 ± 3.0 y; mean height: 180 ± 6 cm). To be included in this study, participants must have had no prior experience with any structured strength training program (i.e., never worked with a personal trainer) and were not taking any medications related to a chronic condition or any supplements to aid in training adaptations (e.g., performance-enhancing drugs like steroids) or any nutritional supplements (e.g., vitamin D, calcium, creatine). Moreover, participants were free from any substantial injuries over the past year and did not suffer from any ailment or conditions for which exercise and maximal exertion efforts may be contraindicated (e.g., chronic low back pain, arthritis, neuromuscular diseases). Specifically, criteria from the American College of Sports Medicine and American Heart Association [20] were used to determine contraindications to aerobic and resistance training/testing, including recent myocardial infarction or electrocardiography changes, complete heart block, acute congestive heart failure, unstable angina, and uncontrolled hypertension. Participants were excluded if they were on a restrictive dietary lifestyle that would require substantial alterations to participant nutritional intake (e.g., vegan, vegetarian) and/or any eating disorders (e.g., bulimia, anorexia).

All participants agreed to participate in this study by signing a consent form. This study was conducted under the Declaration of Helsinki and received ethics approval from the Brock University Research Ethics Board (REB #22-135).

### 2.2. Study Design and Procedures

**Figure 1:**
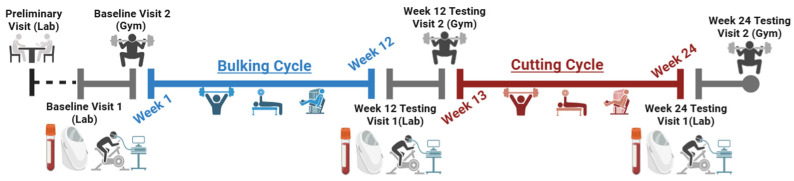
Study timeline (created in BioRender.com).

The pilot study used a longitudinal, repeated measures (within-subject) design where all participants were exposed to the same intervention over a 24-week period, with measurements taken at three specific time points. Participants visited the Applied Exercise Physiology Lab (Brock University) four times; first for a preliminary meeting, followed by three assessment visits, which occurred before the start of the intervention (week 0), at the end of the bulking cycle (week 12), and at the end of the cutting cycle (week 24). The timeline of the study procedures is illustrated in Figure 1. All these visits were scheduled in the morning between 800 and 1100 h to control for any circadian effects.

During the preliminary visit, participants were informed on the purpose, procedures, and risks of the study, and educated on the style of training (i.e., types of exercises, reps/sets) and types/quantities of foods they would be allowed to consume. They also completed questionnaires on their medical history, physical activity, and dietary habits [21].

All assessment visits at weeks 0, 12, and 24 followed the same protocol, starting with a fasted baseline blood draw followed by anthropometric and body composition measurements. Specifically, height was measured with a stadiometer to the nearest 0.1 cm with no shoes, and body composition was measured via air displacement plethysmography (BodPod; Life Measurement Inc., Concord, CA, USA) to get measures of body mass (kg), fat mass (kg), fat-free mass (kg), and percent body fat (%). During these visits, participants also performed an incremental exercise test to exhaustion on the bicycle ergometer (Lode, 911905, Netherlands) to determine their maximal aerobic capacity (VO_2_max). Participants began the test with a 2-min warm-up at a workload of 100 Watts then continued with an incremental 25-watt increase in workload applied every minute until volitional fatigue. Following completion of the test, a 3-min cool-down period was performed. Oxygen consumption (VO_2_) and carbon dioxide production (VCO_2_) were measured continuously with a gas collection system (MAX-II, AEI Technologies, Pittsburgh, PA, USA). Heart rate (HR) was continuously recorded using an integrated HR monitor (Omron HR-310, Omron Electronics, Hoffman Estates, IL, USA). 

In addition to the above measurements, three strength testing sessions were scheduled separately at the Core Health Collective Centre during weeks 0, 12, and 24. During these sessions, participants underwent strength testing using the following exercises: squat, chest press, deadlift, and seated row. The testing was supervised and included several practice sets building up from 40–50% of 1RM and progressively adding weight until 1RM was determined. There were 2–3-min rests between sets. If after 5 sets a 1RM was not determined, then it was calculated using O’Conner’s equation [22] [(1-RM = weight × (1 + (0.025 × reps)]. It should be noted that this method only provides an estimation of 1RM, obtained with a margin of error.

### 2.3. Dietary Protocol

#### 2.3.1. Bulking Cycle (Weeks 1–12)

During the first 12 weeks (3 months), daily caloric intake was determined on an individual basis using the Mayo Clinic Calorie Calculator, which considers age, height, weight, sex, and daily activity levels [23]. Based on their individual daily caloric needs, participants were instructed to consume a slightly hyperenergetic diet (~15% more calories/day than suggested by the Calorie Calculator). Importantly, participants were instructed to consume 25–30% of those calories from protein, 55–60% from carbohydrates, and 15–20% from fats. This breakdown falls within the Acceptable Macronutrient Distribution Ranges (AMDRs) recommended by Health Canada [24]. An emphasis was placed on whole foods; however, sustainability and balance were also taken into consideration. Therefore, an 80/20 rule was employed (i.e., 80% whole foods, 20% foods of personal choice), while still aiming for the daily caloric allotment. To this end, participants were provided with a food scale to measure portion sizes and were educated on how to use the scale and the macronutrient makeup of common foods/beverages. Although not mandatory, to ensure consistency, we recommended the LeanFit whey protein (Coquitlam, British Columbia, Canada) to whoever chose to use a protein powder as part of their increased protein diet. Additionally, participants were provided with a journal to log their daily dietary consumption and were checked on twice weekly to ensure they were adhering to the diet.

#### 2.3.2. Cutting Cycle (Weeks 13–24)

During weeks 13–24, participants’ daily caloric intake was reduced initially by 200 calories from that consumed during the bulking cycle with the same breakdown of nutrient intake (25–30% protein, 55–60% carbs, and 15–20% fats). They were weighed each week aiming to lose a ½ pound of body mass per week. If this was achieved, they stayed on the same 200-calorie deficit. If, however, a ½ pound was not lost over the week, an additional 200 calorie deficit was implemented. These weekly check-ins and calorie adjustments were maintained for the entire 12 weeks. There was no restriction on water intake. Participants were provided with a food scale to measure portion sizes and be educated on how to use the scale and the macronutrient makeup of common foods/beverages. Additionally, participants were provided with a journal to log their daily dietary consumption and were checked on twice weekly to ensure adherence to the diet and address any concerns they may have had.

#### 2.3.3. Dietary Assessment

Participants recorded all food and beverages throughout the entirety of this study using an app or dietary journal of their choice. We recommended participants use the MyFitnessPal application (San Francisco, CA, USA) as it is free and easy to use. Food records were then shared and analyzed for 7 consecutive days during both the bulking and the cutting cycles, and percent adherence to each cycle was calculated using these records [25,26]. Diet adherence was calculated for each participant using the following equation: 7-day recorded calorie sum/7-day target calorie sum × 100.

### 2.4. Resistance Training Sessions

This study consisted of a novel resistance training intervention designed for the study to include three resistance training phases (muscle hypertrophy, muscle strength, and muscle endurance), as described below. The training was scheduled twice per week, on non-consecutive days, at the Core Health Collective Centre (St. Catharines, ON, Canada). All training sessions were led and supervised by a trainer and included three distinct resistance training phases (each 4 weeks in duration) during both the bulking and cutting dietary cycles for a total of 24 weeks. Throughout the 24 weeks, an emphasis was placed on compound exercises, including squats, deadlifts, lunge variations, bench presses, overhead presses, pull-ups, and row variations. As a secondary consideration, accessory exercises such as bicep curls, triceps pushdowns, lateral shoulder raises, calf raises, and core work were also included. Each workout over the 24-week program was a full-body workout and included 3 compound movements followed by 2–3 accessory movements based on the trainer’s discretion (e.g., fatigue level of the participant). Since participants had 2 training sessions/week, workouts were designed in a manner where individuals performed one hip or knee dominant movement on alternating days (e.g., Workout #1 = squats, Workout #2 = deadlift), an upper body horizontal or upper body vertical pushing movement on alternating days (e.g., Workout #1 = dumbbell press, Workout #2 = overhead press), and a compound pull (e.g., seated cable row, pull-ups) each workout. There was no emphasis placed on the order of the exercises, although compound movements were performed before the accessory ones. In terms of the structure of the sessions, these involved groups of 1–3 participants at a time being led through the training and were scheduled based on participant availability.

As mentioned above, the intervention involved three resistance training phases. The first phase was a hypertrophy phase, initially meant to familiarize participants with various exercises and correct form. Participants performed 3 sets of 8–12 repetitions of every exercise (a total of 5–6 exercises) starting with a safe baseline load. As the weeks proceeded, the goal was to progressively overload, and an emphasis was placed on increasing the load for each exercise. The second phase of the training was a strength phase, where a greater focus was placed on eliciting maximal strength. Participants executed 3–5 sets of 3–6 repetitions per exercise (i.e., more sets, lower reps). The exercises selected for this cycle were tailored for maximal strength efforts and included bilateral movements where more weight could be lifted (e.g., squats, barbell bench press). The third phase of training was a muscle endurance phase, with an emphasis on more supersets (i.e., performing multiple exercises consecutively with a very short rest period). Participants performed 3 sets of 15–20 reps for each exercise to increase heart rate and blood flow to the muscle. During this phase, participants may have been asked to perform hip and knee dominant movements in the same workout (e.g., super-setting squats and kettlebell swings) or even combine push and pull movements in a superset. Details about the objectives and intensity of each phase are presented in Table 1. Finally, participants were asked to abstain from any strength exercise/training outside of that conducted for the study. Compliance with the 24-week training program was calculated as [training sessions completed/total training sessions] × 100.

### 2.5. Blood Analysis

Blood samples were collected after an overnight fast of 10–12 h from the antecubital vein at three different time points: at baseline (week 0), the end of the bulking period (week 12), and the end of the cutting period (week 24). For each sample, 10 mL of blood was collected. Blood samples were centrifuged at 3000 rpm for 10 min at 4 °C and the serum was aliquoted into air-tight Eppendorf tubes (Axygen MCT-150-C, Corning Inc., Reynosa, Mexico) and stored in a locked freezer at −80 °C until analysis.

Blood lipids, including triglycerides, total cholesterol, and HDL were measured in serum using a standard lipid panel, and low-density cholesterol (LDL) was calculated using the Friedewald equation [27] by a diagnostic laboratory (LifeLabs, Toronto, ON, Canada). The circulating concentrations of pro-inflammatory cytokines, including TNF-α and IL-6, and the anti-inflammatory IL-10 were analyzed in serum using a commercially available ELLA kit (cat. # SPCKE-PS-010895; ProteinSimple, San Jose, CA, USA). This method analyses each sample in triplicate. All samples were run using Simple Plex software Runner and Explorer (v.3.7.1.12, Bio-Techne, Minneapolis, MN, USA) and analyzed in one single plate. The intra-assay coefficients of variation (%CV) for TNF-α, IL-6, and IL-10 were 2.54%, 4.6%, and 6.69%, respectively.

### 2.6. Statistical Analysis

The data were assessed for normality using the Kolmogorov–Smirnov test, and z-scores for skewness and kurtosis. We used repeated measures one-way analysis of variance (ANOVA) to examine changes in all variables across time (i.e., week 0, week 12, week 24). In the case of a significant main effect for time, post hoc comparisons using paired *t*-tests were performed. Effect sizes are reported as partial eta squared (_p_η^2^) for ANOVA and Cohen’s d for significant post hoc comparisons and were interpreted based on Cohen’s criteria for _p_η^2^: 0.01–0.05 = small, 0.06–0.13 = moderate, ≥0.14 = large effect; and for d: 0.2–0.49 = small, 0.5–0.7 = medium, 0.8 and over = large effect [28,29]. Data are reported as means ± standard deviation. Statistical significance was set at *p* ≤ 0.05 and performed using IBM SPSS Statistics 28 (SPSS Inc., Chicago, IL, USA).

## 3. Results

### 3.1. Feasibility of the Intervention

**Figure 2:**
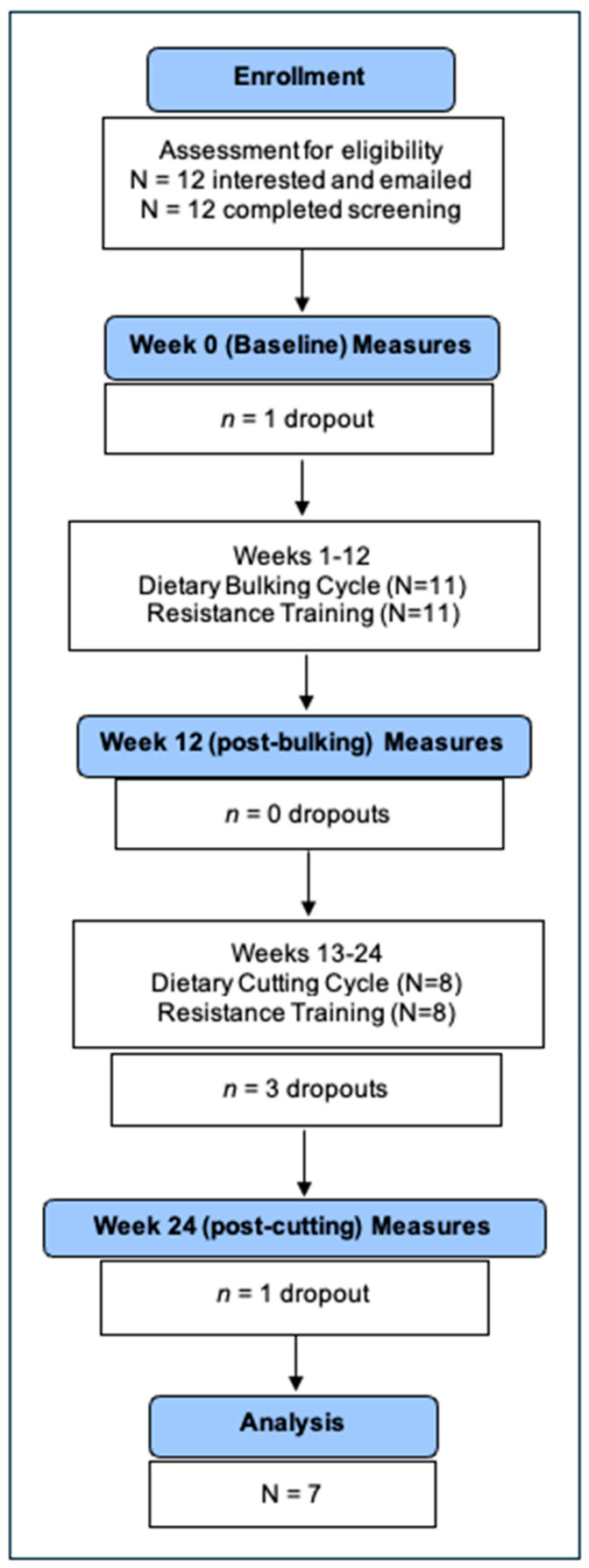
CONSORT study diagram.

Of the 11 participants who started the intervention, three dropped out of the study during the cutting phase, two due to relocation, and one for reasons unknown. One participant completed the 24 weeks of intervention but missed the last post-cutting assessment for reasons unrelated to the intervention. Therefore, a total of seven participants completed the 24-week intervention, attended all three assessments, and were included in the study, resulting in a retention rate of 64%. Figure 2 illustrates the study enrollment.

At baseline, participants were classified as having obesity (BMI ≥ 30 kg/m^2^), with a mean BMI of 35.0 ± 4.6 kg/m^2^. Compliance with the 24-week resistance training program among the seven participants was 96.7 ± 4.3%. Adherence to the 12-week bulking and 12-week cutting cycles of the dietary regimen was 93.7 ± 5.1% and 94.5 ± 3.0%, respectively.

### 3.2. Efficacy of Resistance Training

Strength significantly improved over time, with large effect sizes observed (Table 2). The deadlift and squat exercises both showed significant main effects for time (F = 46.7, *p* < 0.001, _p_η^2^ = 0.90; F = 44.0, *p* < 0.001, _p_η^2^ = 0.90, respectively), with increases from baseline to week 12 testing and then plateaus, such that week 24 values were still significantly higher than baseline, but not significantly higher than week 12 values. Specifically, the weight of the deadlift showed a progressive 40% increase from baseline to week 12 (*p* < 0.001, d = 2.70), with 24-week values 46% higher than baseline (*p* < 0.001, d = 3.21). The same was observed for the squat, which showed a considerable 56% increase from baseline to week 12 (*p* < 0.001, d = 2.55), with 24-week values reaching 65% above baseline (*p* < 0.001, d = 4.44). The bench press and seated cable row exercises also showed significant main effects for time (F = 22.39, *p* < 0.001, _p_η^2^ = 0.82; F = 22.2, *p* < 0.001, _p_η^2^ = 0.82, respectively), with significant increases from baseline to week 12, and then continued significant increases from week 12 to week 24. Specifically, bench press weight showed a significant 15% increase from baseline to week 12 (*p* = 0.004, d = 1.68) and a further 6% significant increase from week 12 to week 24 (*p* = 0.018, d = 1.42), reaching a 22% (*p* = 0.02, d = 1.88) overall improvement from baseline. Likewise, weight lifted in the seated cable row significantly increased by 23% from baseline to week 12 (*p* = 0.001, d = 2.12) and a further 5% significant increase from week 12 to week 24 (*p* = 0.013, d = 1.54), reaching a 28% overall improvement (*p* = 0.005, d = 1.95) from baseline (Table 2). 

As shown in Table 2, we found no significant main effect for time for absolute VO_2_max (F = 1.5, *p* = 0.27, _p_η^2^ = 0.23) or VO_2_max adjusted for FFM (F = 0.39, *p* = 0.69, _p_η^2^ = 0.07).

### 3.3. Body Composition

There was a significant main effect for time for body mass (F = 5.2, *p* = 0.02, _p_η^2^ = 0.46), which significantly increased from baseline to week 12, i.e., post-bulking (+3%, *p* = 0.01, d = 1.32) and then significantly dropped from week 12 to week 24, i.e., post-cutting (−3%, *p* = 0.02, d = 1.22), returning to baseline value (Table 3). Fat-free mass also showed a significant main effect for time (F = 14.9, *p* < 0.001, _p_η^2^ = 0.71), with a significant 4% increase from baseline to week 12 (*p* = 0.003, d = 1.76), and then a plateau, such that week 24 values were still 3% higher than baseline (*p* = 0.004, d = 1.50), but not significantly different than week 12. Although fat mass at 24 weeks was about 9% lower than baseline, there was no significant main effect for time (F = 3.2, *p* = 0.76, _p_η^2^ = 0.35). However, and importantly, there was a significant main effect for time for relative body fat (F = 4.0, *p* = 0.046, _p_η^2^ = 0.40), which significantly decreased from week 12 to week 24 (−4%, *p* = 0.03, d = 0.86), reaching values 6% lower than baseline (*p* = 0.01, d = 1.20) (Table 3).

### 3.4. Circulating Lipid Concentrations

**Figure 3:**
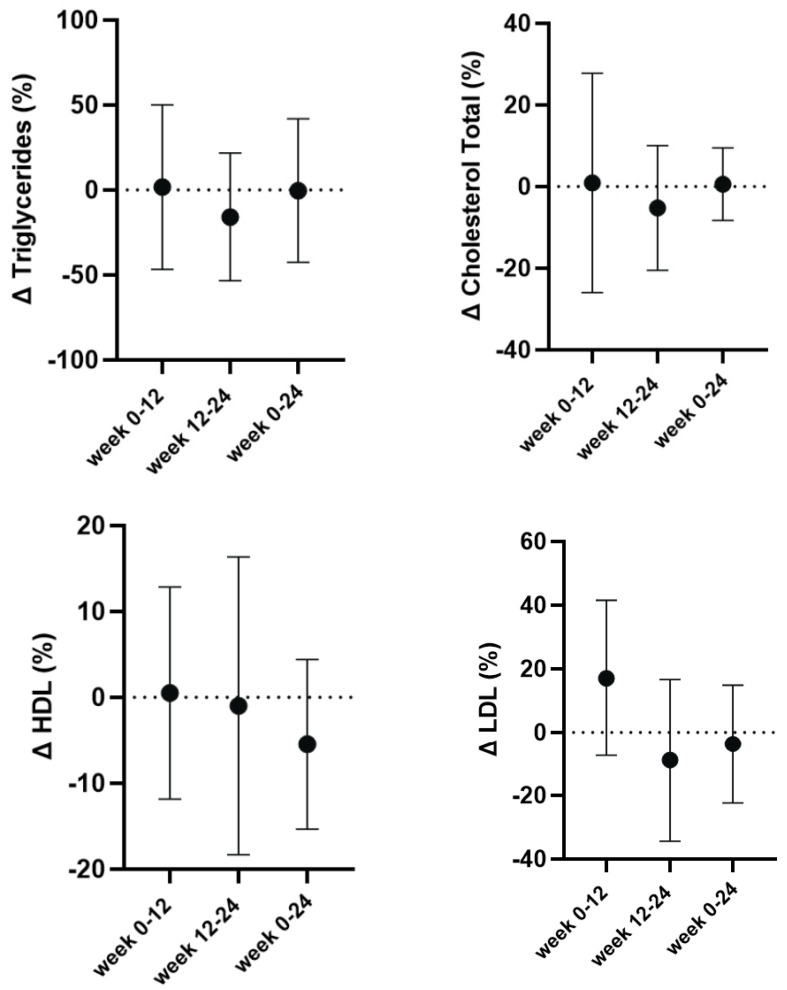
Relative changes in triglycerides, total cholesterol, HDL cholesterol, and LDL cholesterol from baseline (week 0) to post-bulking (week 12) and post-cutting (week 24) cycles in adult males with overweight and obesity (n = 7). Data are expressed as mean ± standard deviation.

The absolute values for lipid concentrations are presented in Table 4. The analysis revealed no significant main effect for time for any of the lipid measures, including triglyceride concentrations (F = 0.81; *p* = 0.46; _p_η^2^ = 0.119), total cholesterol (F = 1.05; *p* = 0.36; _p_η^2^ = 0.15), HDL cholesterol (F = 0.77; *p* = 0.45; _p_η^2^ = 0.11), and LDL cholesterol (F = 1.08; *p* = 0.34; _p_η^2^ = 0.153). These findings suggest that lipid levels remained relatively stable over time. There was also no significant main effect for relative lipid changes (Figure 3).

### 3.5. Circulating Inflammatory Markers

**Figure 4:**
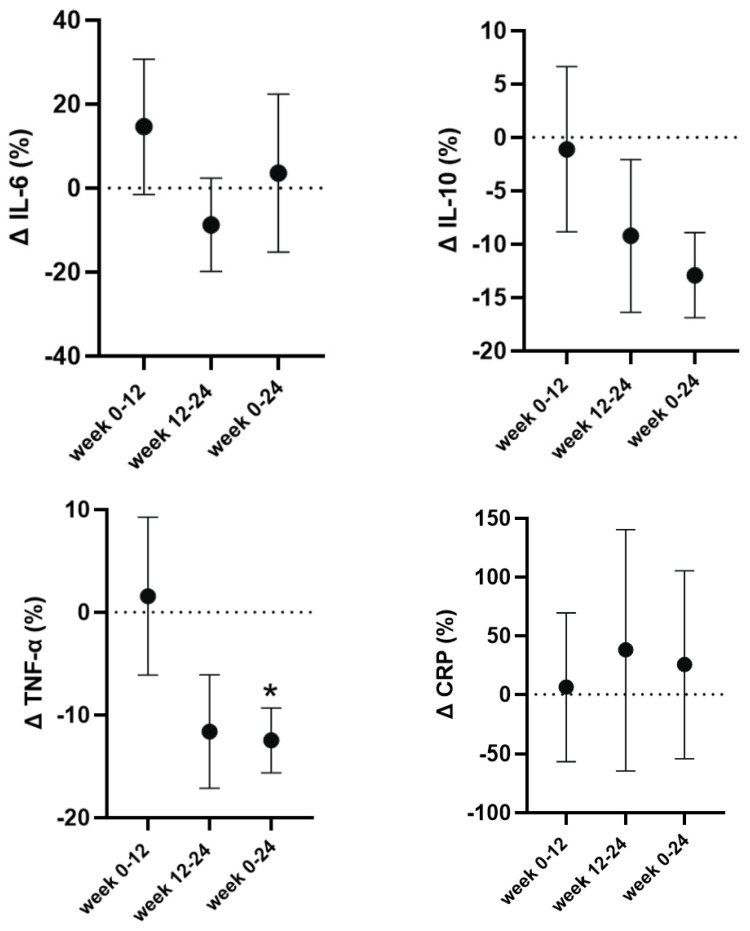
Relative changes in C-reactive protein (CRP), tumor necrosis factor-alpha (TNF-α), interleukin 6 (IL6), and interleukin 10 (IL10) from baseline (week 0) to post-bulking (week 12) and post-cutting (week 24) cycles in adult males with overweight and obesity (n = 7). Data are expressed as mean ± standard deviation. * Denotes a significant difference compared to baseline and week 12 (*p* < 0.05).

The absolute values for inflammatory markers can be found in Table 5. CRP showed no significant main effect for time (F = 0.12; *p* = 0.87; _p_η^2^ = 0.01), and likewise, there was no significant main effect for time for IL-6 (F = 0.55; *p* = 0.59; _p_η^2^ = 0.08) or IL-10 (F = 2.26, *p* = 0.15, _p_η^2^ = 0.27). However, we observed a significant and large main effect for time for TNF-α (F = 3.88, *p* = 0.05, _p_η^2^ = 0.39), which at week 24 (post-cutting) showed significantly lower concentrations compared to baseline (−15%, *p* = 0.008, d = 1.47) and to week 12 (−15%, *p* = 0.04, d = 0.96) (Table 5). To account for the typical individual variability in absolute concentrations, we also present the percent CRP and cytokine changes in Figure 4.

## 4. Discussion

This study introduced a pilot 24-week intervention combining resistance training with a bulk-and-cut dietary protocol in untrained, middle-aged males with obesity. Overall, despite a relatively low retention rate (64%), participants who completed the intervention demonstrated strong compliance and adherence to the 24-week training and dietary cycles, with rates between 93 and 97%. In terms of its preliminary efficacy, the results show that the 24-week intervention led to improvements in body composition, marked by a significant increase in fat-free mass and a decrease in body fat percentage, which was accompanied by significant increases in strength, irrespective of the decrease in caloric intake during the cutting cycle. Additionally, TNF-α had a significant overall decrease from baseline to post-cutting, indicating reduced inflammation. However, no changes were observed in lipids, CRP, IL-6, and IL-10.

### 4.1. Intervention Feasibility

The purpose of this pilot 24-week novel intervention was to tailor a bodybuilding program that combined resistance training with bulking and cutting dietary cycles to be feasible for middle-aged, untrained men. The retention was 64%, which is relatively low and can be attributed to the 6-month long duration and invasiveness of the intervention, with most of the participants dropping out near the end, i.e., during the cutting cycle. Most of the dropouts in this study were due to participants relocating, making the commute to the gym difficult. The compliance to the training for our group of untrained men with obesity was high (96.7 ± 4.3%), and so was the adherence to the bulking and cutting cycles (93.7 ± 5.1% and 94.5 ± 3.0%, respectively). These results can be attributed to the study protocol ensuring that all training sessions were supervised by both a personal trainer and a researcher, leading to not only strong compliance but also ensuring participants were performing all exercises properly and safely. Exercises were taken near or to failure with progressive overload marked by an increase in weight or repetitions to push participants to improve with each session. The dietary protocol was also closely monitored, with weekly checks completed at each training session to ensure participants were recording their meals. If participants fell behind or missed days, we encouraged them to take pictures of all meals and log their food as accurately as possible when they had free time. This constant monitoring and supervision were significant strengths of this pilot intervention.

Given its type and design, this training protocol, which focused on strength with no aerobic component, resulted in significant increases in strength but no significant increase in the absolute VO_2_max or VO_2_max adjusted for FFM. The lack of a significant increase in VO_2_max can likely be attributed to the non-aerobic nature of the training. This aligns with previous resistance training studies in overweight and untrained individuals, which found little to no significant improvements in VO_2_max compared to aerobic or combined training [30,31]. This may also stem from the small sample size, as the absolute VO_2_max seemed to increase by 11% after week 12 (post-bulk) and remained elevated by +8% after week 24 (post-cutting phase). Post-priori power calculations using the observed effect size (_p_η^2^ = 0.23) indicate that a sample size of 11 was required for the increase in VO_2_max to reach significance. However, the impact of our intervention on VO_2_max was reduced when accounting for FFM (_p_η^2^ = 0.07). This outcome is expected considering that FFM increased as a result of the resistance training type of intervention (as discussed below) and the established connection between FFM and VO_2_max [32]. 

### 4.2. Improvements in Body Composition and Strength

Compared to the current literature, which has looked at strictly resistance training or aerobic training and hypocaloric diets, our study showed greater improvements in strength and body composition with only two training sessions per week (compared to three sessions per week) [33,34]. One probable reason for these differences is the consistent monitoring of the training by the personal trainer and researchers who were always present to ensure exercises were performed correctly and close to maximum/failure, thus leading to high compliance. In these two previous studies, compliance was often self-reported by the participants, making it unclear whether they trained safely and effectively [33,34]. Moreover, even if compliance in these studies matched the level achieved in the present study (i.e., >90%) [33,34], it suggests that resistance training twice per week may be as effective as three times per week for untrained individuals. Another possible explanation is that our bulking and cutting diet was more conducive to improvements in body composition and strength, although further research is needed to confirm this assumption.

Although the effects of the dietary bulk-and-cut alone on body composition and strength are unknown, we observed a favorable change in body composition with our combined training and dietary intervention. A major concern for most of the participants was that they may lose strength while eating a hypocaloric diet, yet our resistance training program successfully resulted in increasing strength from the end of the bulking phase to the end of the cutting phase in two of the four exercises. We attribute this outcome to an increase in lean mass following the bulking phase (week 12), which was then sustained after the cutting phase (week 24). Moreover, following the bulking phase, there was a significant increase in body mass, which was expected as participants were instructed to consume more calories. After the subsequent cutting phase, body mass returned to baseline levels, though not without a favorable shift in body composition. That is, by week 24, body mass was similar to baseline levels, yet there was a significant increase in FFM and a notable decrease in body fat percentage. Although resistance training in untrained or overweight individuals leads to improvements in body composition [10,11,35], the effects of overeating or caloric bulking paired with resistance training are largely understudied in this population. Previous studies on varying caloric surpluses have found some evidence suggesting that greater surpluses lead to larger increases in strength and body mass among resistance-trained individuals [36,37]. However, it remains unclear whether such a high degree of overfeeding is necessary for untrained individuals and whether it yields measurable improvements in strength and body composition compared to moderate or maintenance-level diets with adequate protein supplementation.

### 4.3. No Changes in Circulating Lipids

There were no significant changes in triglycerides and cholesterol levels. Previous studies using comparable resistance training protocols alone have demonstrated significant reductions in triglyceride and LDL cholesterol levels [38,39,40,41]. One potential reason for the lipids remaining the same in the current study could be from the diets adapted by participants. Many were inclined towards eating a diet high in carbs and saturated fats to reach caloric goals during the bulking phase. This style of eating can lead to an increase in circulating cholesterol and triglycerides [42,43]. Moreover, the limited studies incorporating time-restricted eating or low-calorie diets alongside resistance training have reported conflicting findings on lipid profile improvements [44,45,46]. As a result, the approach to eating and training in the current study may not be the most effective for improving lipid levels. 

### 4.4. Changes in Resting Concentrations of Inflammatory Markers

Given that CRP levels exceeding 2.0 mg/L are considered a risk-enhancing factor for cardiovascular disease, the CRP concentrations of our male participants with obesity can be classified as slightly elevated [47]. Importantly, CRP did not significantly change across the 24-week intervention. This finding agrees with previous studies using resistance training alone three times per week for nine and 16 weeks in a comparable population, which also reported similar resting CRP concentrations and no training-induced changes [48,49]. Regarding exercise and diet, current research shows aerobic and high-intensity interval training (HIIT) paired with caloric restriction were the most effective at reducing elevated levels of CRP [50,51,52]. In these studies, participants exhibited higher baseline CRP levels, while groups engaging in HIIT and aerobic training without caloric restriction showed inconsistent CRP results.

Regarding the cytokine concentrations, the most notable change was the reduction in TNF-α, which, by the end of the 24 weeks, and after the cutting cycle, reached significance. In addition, IL-6 concentrations, although not significant, decreased post-bulk and then returned near baseline levels post-cut. Previous studies have also reported decreases in the circulating concentrations of major pro-inflammatory cytokines during timed-restricted eating and hypocaloric diets in endurance and resistance-trained individuals [53,54]. However, other studies using intermittent fasting on healthy males have found no significant changes [55]. Increased adipose tissue has also been directly correlated with higher levels of circulating pro-inflammatory cytokines like IL-6 and TNF-α [56,57,58]. This makes it challenging to determine whether the decrease in TNF-α in the current study was due directly to either the loss of fat by our participants, the dietary changes, or the consistent resistance training. However, the significant decrease in circulating TNF-α is an indication of reduced inflammation, which is consistent with the loss of fat and an important factor for individuals with obesity.

In contrast, we observed no significant changes in the anti-inflammatory cytokine IL-10. The current literature presents mixed findings on the effects of exercise training on circulating IL-10 levels. For instance, a decrease in resting IL-10 concentrations was noted in frail older women after a resistance training program [59]. Conversely, a meta-analysis on aerobic, resistance, and combined training in patients with metabolic syndrome reported increases in IL-6 and IL-10 levels following the combined regimen [60]. In cancer patients, exercise training did not significantly affect IL-10 levels [61]. Additionally, a large 12-month randomized controlled trial found no impact of aerobic exercise training on circulating IL-10 concentrations in post-menopausal women [62]. This suggests that the relationship between exercise, diet, and IL-10 remains unclear and may vary based on individual factors and training regimens.

### 4.5. Limitations and Strengths

This was a pilot study, and therefore, it was limited by its small sample size and the absence of a control group. The small sample size cannot be representative of the general population, thus making it difficult to generalize. Post-priori power calculations using G*Power (version 3.1) analysis and an average observed effect size (_p_η^2^ = 0.35) with a power of (1-β) = 0.80, and a probability level of *p* = 0.05, indicate that a sample size of 15 was required to reach significance in other variables. Irrespective of the small sample, however, significant changes were observed in body composition and TNF-α. Furthermore, excluding female participants and having a relatively low retention rate (64%) can indeed impact the generalizability and effectiveness of the intervention, especially in larger groups with obesity and comorbidities. The absence of a control group that would follow the bulk-and-cut dietary protocol without resistance training makes it challenging to isolate the effects of the intervention. Thus, any future study should include a control group. Another limitation was regarding strategies participants used to hit target calories. While we encouraged participants to maintain their usual eating habits, many adopted new strategies for both the bulking and the cutting cycles. Some gravitated towards a Western-styled diet to consume higher calories while others took this as an opportunity to prepare healthier meals, characterized by lean protein, unsaturated fats, and whole grains. Then, for the cutting phase, when calories had dropped substantially, some participants would fast until the evening while others ate smaller meals throughout the day. This variability was not necessarily a limitation but because of the small sample size, it limits the generalizability of these findings. Nevertheless, the results from the current pilot show substantial improvements in body composition and strength even with the inclusion of Western-style food items.

This pilot study has some notable strengths. First, to date, this novel combination of resistance training with the specific bulk-and-cut dietary protocol, has only been practiced by professional bodybuilders to quickly gain fat-free mass and lose body mass, and the current findings show promise in obese men as well. Second, regarding feasibility, feedback from the participants was all positive. Although some admitted the difficulty and discipline it required to follow the program for 24 weeks, the majority mentioned they enjoyed the experience. Lastly, the significant changes in body composition and retention/increase in strength demonstrate that this program can be a viable protocol for overweight or obese individuals.

## 5. Conclusions

Despite a relatively low retention rate (64%), the pilot 24-week resistance training intervention combined with a dietary bulk-and-cut protocol demonstrated strong compliance and adherence and was effective in improving body composition and lowering TNF-α concentrations, but with no effect on lipid profiles, in untrained, middle-aged adult males with obesity. 

## Figures and Tables

**Table 1:** Breakdown of resistance training phases.

Training Phase	Weeks	Rep Range (Set Range)	Intensity (%1RM)	Objective
Hypertrophy	1–4 & 13–16	8–12 (3–5)	Moderate (50–80%)	Familiarization with exercises, correcting form, and progressive overloading. Reaching failure between sets 8 and 12.
Strength	5–8 & 17–20	3–6 (5–6)	High (80–95%)	Maximal strength, incorporating more sets and longer rest periods while working up to a weight to elicit failure between reps 3 and 6.
Endurance	9–12 & 21–24	15–20 (3–4)	Low (<50%)	Muscular endurance, lifting weights with speed and explosiveness. Superset certain movements while limiting rest time between exercises.

**Table 2:** Strength measures and VO_2_max at baseline (mean [$\bar{x}$] ± standard deviation [SD]), after the bulking (week 12), and after the cutting (week 24) cycles (n = 7).

	Baseline ($\bar{x}$ ± SD)	Week 12 ($\bar{x}$ ± SD)	Week 24 ($\bar{x}$ ± SD)
Deadlift (kg)	133 ± 16	187 ± 22 ^α^	193 ± 23 ^β^
Squat (kg)	89 ± 26	140 ± 11 ^α^	146 ± 21 ^α^
Bench Press (kg)	81 ± 13	93 ± 18 ^α^	99 ± 18 ^α,β^
Seated Cable Row (kg)	87 ± 16	107 ± 9 ^α^	112 ± 9 ^α,β^
VO_2_max (L/min)	3.14 ± 0.69	3.49 ± 1.05	3.40 ± 1.04
VO_2_max (mL/kgFFM/min)	44.1 ± 5.3	46.5 ± 8.2	45.8 ± 8.2

α = significant differences from baseline (<0.05); β = significant differences from week 12 (*p* < 0.05).

**Table 3:** Body composition measures (mean [$\bar{x}$] ± standard deviation [SD]) at baseline, after the bulking (week 12), and after the cutting (week 24) cycles (n = 7).

	Baseline ($\bar{x}$ ± SD)	Week 12 ($\bar{x}$ ± SD)	Week 24 ($\bar{x}$ ± SD)
Body Mass (kg)	113.5 ± 14.9	117.0 ± 16.1 ^α^	113.6 ± 15.5 ^β^
Fat-Free Mass (Kg)	71.9 ± 8.3	74.8 ± 8.6 ^α^	74.3 ± 8.7 ^α^
Fat Mass (Kg)	41.6 ± 10.4	42.2 ± 9.3	39.3 ± 8.9
Relative Body Fat (%)	36.3 ± 5.5	35.8 ± 3.9	34.3 ± 4.2 ^α,β^

α = significant differences from baseline (<0.05); β = significant differences from week 12 (*p* < 0.05).

**Table 4:** Absolute lipid concentrations (mean [$\bar{x}$] ± standard deviation [SD]) at baseline, after the bulking (week 12), and after the cutting (week 24) cycles (n = 7).

	Baseline ($\bar{x}$ ± SD)	Week 12 ($\bar{x}$ ± SD)	Week 24 ($\bar{x}$ ± SD)
Triglycerides (mmol/L)	2.03 ± 1.03	1.82 ± 0.9	1.90 ± 0.97
Cholesterol Total (mmol/L)	5.46 ± 1.54	5.49 ± 1.54	5.83 ± 1.00
HDL Cholesterol (mmol/L)	1.22 ± 0.25	1.21 ± 0.20	1.13 ± 0.26
LDL Cholesterol (mmol/L)	3.41 ± 0.78	3.87 ± 0.6	3.67 ± 1.02

**Table 5:** Absolute concentrations of C-reactive protein (CRP), tumor necrosis factor-alpha (TNF-α), interleukin-6 (IL-6), and interleukin-10 (IL-10) concentrations (mean [$\bar{x}$] ± standard deviation [SD]) at baseline, after the bulking (week 12), and after the cutting (week 24) cycles (n = 7).

	Baseline ($\bar{x}$ ± SD)	Week 12 ($\bar{x}$ ± SD)	Week 24 ($\bar{x}$
CRP (mmol/L)	1.80 ± 1.15	1.75 ± 1.16	1.63 ± 0.47
TNF-α (pg/mL)	16.05 ± 2.47	16.14 ± 3.30	13.94 ± 1.63 ^α^
IL-6 (pg/mL)	2.27 ± 0.61	2.46 ± 0.63	2.11 ± 0.49
IL-10 (pg/mL)	2.59 ± 0.54	2.50 ± 0.44	2.25 ± 0.48

α = significant differences from baseline and week 12 (*p* ≤ 0.05).

## Data Availability

The data supporting the findings of this study are available from the corresponding author (P.K.) upon reasonable request due to ethics regulations.

## Abbreviations

The following abbreviations are used in this manuscript:

RT	Resistance Training
HIIT	High Intensity Interval Training
CRP	C-Reactive Protein
IL-6	Interleukin-6
IL-10	Interleukin-10
TNF-α	Tumor Necrosis Factor-alpha
LDL	Low-Density Lipoprotein
HDL	High-Density Lipoprotein
1RM	1 Repetition Maximum
AMDR	Acceptable Macronutrient Distribution Ranges
FFM	Fat-Free Mass
BMI	Body Mass Index
ANOVA	Analysis of Variance
